# Guideline-informed care for osteoarthritis: Support needs of community pharmacists and healthcare professionals in Nigeria, West-Africa

**DOI:** 10.1016/j.ocarto.2025.100678

**Published:** 2025-09-04

**Authors:** Oladapo Adetunji, Ibidunni Alonge, Ebunoluwa Ayinmode, Tolulope Owoyemi, Adebimpe Ogunbanjo, Simon White, Adewale Adebajo, Christian Mallen, Krysia Dziedzic, Opeyemi O. Babatunde

**Affiliations:** a University of Ibadan, Ibadan, Nigeria; b West African Institute for Applied Health Research, Ibadan, Nigeria; c Pharmacy Division, Lagos State Health Service, Lagos, Nigeria; d Keele University, School of Pharmacy and Bioengineering, Keele, Staffordshire, UK; e School of Medicine and Population Health, University of Sheffield, UK; f School of Medicine, Primary Care Centre Versus Arthritis, Keele, Staffordshire, UK; g Impact Accelerator Unit, Keele University, Keele, Staffordshire, UK

**Keywords:** Osteoarthritis, Care pathways, Community pharmacy, Primary care, Nigeria, West-africa

## Abstract

**Objective:**

The study examined the context, training needs, and extent to which community pharmacists in Nigeria have the knowledge, resources, and capability to manage OA in line with evidence-based recommendations.

**Methods:**

Focus group discussions (n ​= ​2) were conducted. Discussions explored current practice, the support needs, and perceptions of a new OA care model where pharmacists could be trained to screen, educate, and refer patients. Data was analyzed thematically, including stakeholder workshops to aid interpretation.

**Results:**

Data from two focus groups with 22 healthcare professionals revealed five key themes. Community pharmacies were the first point of call for most patients. Care pathway and navigation onwards were influenced by patient affordability. Radiological examinations and blood tests underpinned diagnosis and care predominantly involved pharmacological approaches. There was no local care pathway agreed/established for health professionals, and multidisciplinary team collaboration/care for OA was limited. Participants expressed the need for professional education and the development of national guidelines to inform osteoarthritis management in primary care.

**Conclusions:**

Nigeria's OA care pathway is fragmented, involves overuse of medication, and has limited access to non-pharmaceutical treatments. A unified, evidence-based approach with adequate training and multidisciplinary collaboration is essential for effective primary care and reducing health inequalities.

## 1 Background

The global burden associated with osteoarthritis (OA) and joint pain is increasing in Africa and most low- and middle-income countries (LMICs) [[1], [2], [3]]. Compared to High-income countries, Additionally, OA disproportionately affects younger populations in LMICs given that many people work in high-risk industries (such as manufacturing, and farming), where preventative safeguards and health and safety standards may be suboptimal [4]. Yet, there is little evidence of research prioritizing effective implementation of OA management programs in LMICs.

OA not only causes significant problems for individuals and their families but also has a major economic and societal impact through time lost from work, lost income, and reduced productivity [5]. Decreased quality of life, difficulty navigating healthcare systems, increased healthcare costs, and socio-cultural contexts frequently drive patients to try unproven approaches to pain management outside primary care and to seek help from non-professionals [6]. In Africa, particularly Nigeria, community pharmacies and drug retail outlets are easily accessible, without the need for a prior appointment and usually without the need for consultation charges.

Community pharmacy has the potential to be an important resource to help people with OA optimize the use of medicines for pain management. Internationally recognized guidelines highlight non-pharmacological approaches (such as exercise, weight management, diet modification), as core to the management of OA [7]. However, pharmacological approaches appear to be overutilized in West Africa, partly due to cultural beliefs, access to over-the-counter medicines, limited knowledge and skills by healthcare professionals, and lack of synergy and awareness of evidence-based recommendations among care professionals [8].

Furthermore, community pharmacists often remain the only continuous source of long-term help and advice for many patients with OA and joint pain. Many people never receive a formal diagnosis or care in health settings [9]. Existing opportunities can be leveraged to improve OAcare; however, it is crucial to investigate current perceptions and care pathways and to determine how best to support healthcare practice in line with core guideline recommendations. Therefore, this study aimed to explore the current management practices of community pharmacists and healthcare professionals and understand their support needs to inform effective implementation of guideline-informed OAmanagement program in Nigeria, West Africa.

## 2 Methods

### 2.1 Study design

The current study was part the first phase of a wider research study and pilot implementation project in Nigeria aimed at enhancing evidence-based care for people with OA in West Africa - Joint Implementation of Guidelines for Osteoarthritis in West Africa: JIGSAW-A (https://jigsawafrica.com) [10,11]. To contextualise existing resources prior to feasibility testing, this phase of JIGSAW-A project, aimed to explore the current management practices of community pharmacists and healthcare professionals and understand their support needs to inform effective implementation of guideline-informed OAmanagement program. Ethical approval has been granted by the University of Ibadan, Research Ethics Committee and Keele University FMHS Research Ethics committee (UI/SSHEC/2022/0023; KU0420). The procedure for performing and reporting this research was in line with the consolidated criteria for reporting qualitative research (Coreq, 2007) [12]. Focus Group Discussion (FGD) was employed to provide information on the perception of community pharmacists, physiotherapists, and other healthcare professionals about OA, existing care pathway, health professional roles in managing OA, barriers to optimal management, and recommendations for improving care. The FGDs facilitated through interactive dialogue were used to obtain in-depth information about the healthcare professionals’ opinions and thoughts on management of OA.

### 2.2 Recruitment and sample size

Community pharmacists, physiotherapists, pharmacy technicians, nurses and medical doctors (orthopedic surgeons and family physicians) were recruited through broad advertisement via a snowballing technique including via professional organization networks (e.g., association of community pharmacists, Nigerian society of Physiotherapy, WhatsApp groups, and professional contacts (via email) of study team members to relevant contacts. To provide an appropriate range of depth and insight, a sample of 30 healthcare professionals were sought for this study. From those that expressed interest, participants were purposively selected to represent diverse socioeconomic communities and a range of practice/pharmacy business models in Nigeria. The design features are congruent with recommended approach with a focus on generating rich discussions [13] and from a wide range of perceptions and experiences via up to three focus groups.

### 2.3 Eligibility criteria

Qualified healthcare professionals practicing in community/primary/state healthcare settings in Nigeria, who have cared for >50 patients living with OA within the last six months. This was mainly to ensure that participants are familiar with the subject of osteoarthritis and joint pain and can participate fully in the FGD. We excluded hospital-based healthcare professionals who within their role do not usually attend to osteoarthritis patients or have not provided care for patients with OA within the last six months.

### 2.4 Data collection procedure

This study followed ethics regulations as approved by the Research Ethics Committees of the University of Ibadan, Nigeria, and Keele University, United Kingdom (UI/SSHEC/2022/0023; KU0420).

An FGD guide (box 1) was developed a priori based on literature reviews, contextual knowledge of the study team on OA management and healthcare in Nigeria [[14], [15], [16], [17], [18]]. The areas of focus/themes were healthcare professionals’ knowledge of OA, pathway of care for OA, role of community pharmacists in the management of OA, challenges and support needs for managing OAin line with core guideline recommendations in West-African health settings. FGDs were conducted in person and as hybrid (physical and virtual), with each session lasting ​∼ ​90 ​min on average (excluding time for introductions and familiarizations). The 2nd FGD was conducted as an “hybrid” focus group to remove barriers to participation. Most participants (n ​= ​8) were online, and the in-person participant was in the room with researchers. The data collection process was facilitated by experienced researchers (OA, OB) with subject expertise, trained in the sensitivity and conduct of focus groups. OA is a Pharmacist and OB – a physiotherapist), both have experience of practice within the study settings, have complementary skills set and committed to multiple realities. A scribe was present to record field notes, including nonverbal gestures and group dynamics.

Box 1 **JIGSAW-A FGD Topic Guide**

• **Introduction**: Welcome, explanation of JIGSAW-A project, and FGD study, informed consent, demographic data, confidentiality
• **Topic 1**: Current practice, management, and pathways for delivery of care for OA
• **Topic 2:** Views and perspectives about proposed new model of care with enhanced role for community pharmacies and multidisciplinary team working
• **Topic 3:** Expressed support needs to aid delivery of proposed model of care
• **Topic 4:** Potential challenges and perceived facilitators of optimal OA care delivery
• Any other points to discuss
• **Summary** of discussion, thanks, and permission for further contact

Alt-text: Box 1

### 2.5 Data analysis

All focus groups were audio-recorded, translated, and transcribed verbatim by professional services and checked against recordings for accuracy by core research team members who facilitated the FGDs. Field notes taken during FGDs were reviewed as means of cross-checking data in the transcripts. transcripts were identified, manually coded, and analyzed using thematic analysis [19,20]. It has been shown to be an effective method for categorizing concepts, developing themes, and conceptualizing connections and/or threads across a set of qualitative data. Open, sentence-by-sentence coding was performed by naming or defining concepts through close examination of the FGD data. Provisional categories of themes and sub-themes were developed simultaneously or after re-reading the transcripts. As recommended by Strauss and Corbin, coding procedures were performed in sequence immediately after each focus group was conducted, this further directed and refined the conduct of the subsequent FGD [21]. Each of the focus group transcripts was first analyzed separately to capture distinctive contexts, key issues, and themes, which were coded and categorized, and a comparison was made across the transcripts to look for similarities and differences [22]. Data coding and analysis were conducted by pairs of two independent researchers (OB & TO; OA & OB) and subsequently involved wider members of the research team, in consensus on definition of themes, guided by the process outlined by Braun & Clarke, 2006 [23]. A methodology suitable for identifying and organising shared experiential themes in qualitative data. An interpretivist perspective was used to develop understanding of participants' reality and context of their professional practice while acknowledging subjectivity of the researchers.

### 2.6 Public involvement and engagement

The study team worked together with 2 male public contributors (≥65 years) with lived experience of OA (multiple joint pain hip and knee, >5 years) who contributed to key aspects of the design, and interpretation of findings in this study. As part of a wider program of work, public contributors received training and guidance a priori about being involved in research. Training was over two sessions covering the aims of the project, principles of public involvement in research and the power of their lived experience in shaping research. Contributors were also supported with tailored training materials and a dedicated assistant to support their participation and encourage meaningful engagement in project meetings. Specifically, for this study, they commented on draft focus groups topic-guides highlighting the need to discuss inter-professional links, communication and referral for OA care. Meetings were held with public contributors in person, and they were free to contact researchers with additional comments via telephone conversations. Furthermore, a stakeholder meeting with the two public contributors and three health professionals and representatives of professional bodies were held to aid interpretation and to address consonance and dissonance within the data.

## 3 Results

Two focus groups involving 22 healthcare professionals were conducted (see Table 1 for summary of study demographics). The FGD involving pharmacists (FGD/CP) included 5 males, 6 females: one pharmacy technician, two consultant pharmacists, seven community pharmacists, and one hospital-based pharmacist with more than ten years working experience as a community pharmacist. Participants practiced in a mix of urban (75 ​%) and rural (25 ​%) areas with years of practice ranging between 5 and 39 years (mean 21.5 years). The second FGD was conducted with multidisciplinary healthcare professionals and comprised two nurses, one orthopedic doctor, one family physician, three physiotherapists, and four pharmacists. The years of practice ranged from 5 to 45 years (mean, 8 years). In the FGD involving pharmacists, participants reported that they regularly attended to between 18 and 24 patients (aged 45–90 years) with OA per week on average. Average number of patients with OA were more for the 2nd FGD (∼37 per week). Typical patients who consult with the study participants (across both FGDs) for joint pain varied from low-middle-high income earners, retired pensioners and unemployed middle aged.

**Table 1:** Focus group discussion demographics.

Group	Number of participants	Gender Ratio (M:F)	Profession(s)	Years of Practice (Range/Mean)	Location of Practice	OA Patient per week on a average
FDG/CP	11	5:6	2 consultant pharmacists, 7 community pharmacists, 1 hospital-based Pharmacist (ex-community), 1 pharmacy technician	5–39 years (mean: 21.5 years)	75 ​% urban, 25 ​% rural. Pharmacists from Ibadan and Lagos	18–24
FDG/HCP	9		2 nurses, 1 orthopedic doctor, 1 family physician, 2 physiotherapists, 3 pharmacists	5–45 years (mean: 8 years)	Broader HCP perspective on OA management in community, primary & Secondary care settings	4–70

Community Pharmacists (CP).

Health Care Practitioner (HCP).

Focus Group Discussion (FDG).

**Table 2:** Overview of the themes with associated participant quotes.

Theme	Sub-theme	Quote
Assessment and diagnosis of osteoarthritis	Investigations and referrals	*“Most time we do referral to the specific hospital, or nearest hospital that we know the patient can make use of …“.***P5/FGD/HCP** *“I will say basically for me, it is not about diagnosing, when patients are referred from the hospital because I belief that as pharmacist, I do not have all that is required for doing the best work on that …“.***P6/FGD/HCP** *“We do our own medical diagnosis. I guess the assessment is really subjective, that is when we want to: What is the history of this patient? How did the patient start experiencing some of the symptoms he or she started experiencing. We do different components for each person. So going to the objective assessment, where we want to check for other objective value and able to assess for functionally how some of these symptoms have affected them”.***P2/FGD/HCP**
Care navigation and current management of osteoarthritis.	Usual care pathway	*“The first thing is based on the patient's complain … it's usually based on the clinical symptoms that the patients complain, that's the basic, only a few of them you refer to the lab to go and do x-ray and the rest, but usually it is based on the clinical presentation that the patients presented”.***P7/FGD/CP** *“Okay, people that are, like he said, people that are specialized in bone care, are the best, well, that does not mean that pharmacies cannot play a role because they are like the first point of call, so when the patient comes, the patient drops the complains, I'm having pains here, you give analgesics to help relieve the pain, then you can place bone supplements like autocade, bone care, joint relieve and the rest, then you monitor the patient, if there is no relief, for like a week or two, then you refer to orthopedics that can manage the patient properly”.***P1/FGD/CP** *“Usually they are referred from a place like xx, where probably they would have been seen by an Orthopedic doctor .., I am speaking as a physiotherapist and a consultant”.***P4/FGD/HCP** *“You know I mentioned that you will first consider the sign and the symptoms that the patient presented with, you take your history, you examine the patients, you ask the patient to carry out some investigations, so let raise two scenarios, So if Mr. A comes and at the end of the day, you do full blood count, BSI, rheumatoid factor and the most important investigation, the radiological investigation, so if the x-ray shows a mild form of OA that can be managed on medication and the other non-pharmacological treatment, especially, if the patient is overweight, you go on diet modification to reduce their waste, so as a doctor, you also inform them of what to eat, what not to eat, how to increase their physical activity that will not further worsen their condition, to the best of our knowledge, we tell them what we know, but for further management, because of the kind of environment where I am, we have access to all this specialty to refer”.***P2/FGD/HCP**
	Pharmacological approach	*“… effective medications, they don't come cheap; they don't come cheap; you find out these patients can hardly even buy food for themselves, talk less of ermm, thinking of bringing out money from their pocket to buy medications”.***P11/FGD/CP** *“Then another important factor is the severity of the pain and the location of the severity. When you now get all this, you now be able to advise the patient better, so that is my take …“***P4/FGD/HCP** *“But basic line should be provided. Just like we know that other vital signs are important. Let us also ensured that pain is considered, because level of pain is usually a pointer to the kind of management that should be given … You see a patient, the patient pain is, I mean severe, excruciating, will usually send them back to the clinic, even to the medical doctor immediately. Let us find a way of lowering this pain first, because, if the pain is so high, and it is not addressed, no matter the kind of full treatment of a good therapy you may want to render, the person won't be able to endure, it is part of strategy will are adopting then and it is still the same practice most general hospital adopt based on the level of pain”.***P5/FGD/HCP** *“… You want to relieve pain and as much as possible, improve the quality of life of the patients. For instance, if it is the knee, mobility is what the patient desires, so you want to give medications that can improve on the mobility and reduce the inflammation; you also want to be very careful so that you don't give drugs that will create other problems for the patients, and it is also very important to know where to draw the line. Some of this arthritis or osteoarthritis that you see, there might be some deeper investigations that ought to be done, so that you don't just look at them superficially. The moment you start the medication, and the patient is not responding, then you know you have to refer the patient”.***P11/FGD/CP**
	Non-pharmacological approach	*“… And then in my own cases, most times, the patients I have, they've already known they have arthritis, maybe they've been diagnosed, the pain would, you know, consistent coming, buying pain drugs, why are you buying these things all the time, further questioning, you get to know that they are; they have knee pains, and then some from the hospitals, they've already been diagnosed already, so they just come for refilling. And then what I noticed is that most arthritis cannot be totally cured but managed. Manage it as far as the pain is reduced and the patient can have good quality of life” …***P1/FGD/CP** *“if it is the knee, mobility is what the patient desires, so you want to give medications that can improve mobility, reduce the inflammation****”. P1/*FGD/CP** *“It is known that it is a combination of pharmacological and non-pharmacological management, in order to reduce the pain, and to give the patient good quality of life … ideally educate the patient, about some lifestyle adjustment, which includes proper diet, that means low salt, low fat, negligible sugar, taking a lot of fruits and vegetables, taking a lot of water, then you do the of the weight and height of the patient, you asses the BMI …”* *“… By the time you as a GP, as general practitioners … you need to have a lot of health education and promotion for the patient, the care givers, families …“***P7/FGD/HCP**
Multidisciplinary care and community pharmacies as first point of call.	First point of call	*“Pharmacies are like the first point of call, so when the patient comes, the patient drops the complains, I'm having pains here, you give analgesics to help relieve the pain, then you can place bone supplements like autocade, bone care, joint relieve and the rest, then you monitor the patient, if there is no relief, for like a week or two, then you refer to orthopedics that can manage the patient properly”.***P1/FGD/CP** *“… they will still go back to the pharmacist in their community because they are closer to them, and all the bottle neck of dropping your card, getting your case note, do this, do that, going up and down is remove d. So that is while the community pharmacists are very (very) critical. They have a critical role when it comes to patients' management in our environment”.***P7/FGD/HCP**
	Multidisciplinary collaboration	*“Okay, thank you, I believe they should be involved from the word go? It is a teamwork and every aspect; every member of the team is equally important. So, I believe that from the ward role, from the set role they should be involved, that is my take on that”.***P3/FGD/HCP**
Barriers to management of osteoarthritis.	Poor collaborative care	*“… the urban community pharmacists, they do not have much time, they are not easily accessible, some are busy in their office dispensing drugs etc., doing all of those things”.***P8/FGD/HCP** *“The other aspect I want to mention is the aspect of making sure that we have a two way understanding of having collaborative care, such that when patient are(are, are) referred and they are also referred back to the pharmacists that will be an encouragement for them and for me …”***P6/FGD/HCP**
	Underlying health ailments	*“It has been the same, we have challenges when it is non-steroidal, but the challenges of ulcer will take us to something like arthrotec, which is misoprozone, diclofenac, and it is very common that most of the patients with arthritis, osteoarthritis, are also having ulcer or something like that, it has been a very big challenge to get other drugs beside arthrotec that will really work; they claim others also they are astruslective … in which most of the time they will still complain, that is the extra challenges”.***P3/FGD/CP**
	Knowledge barrier	*“… then at the end of the day, the patient end up being with community health workers or the extension workers or auxiliary nurses and their level of knowledge when it comes to patient care like this is very negligible”.***P2/FGD/HCP**
	Funds for healthcare workers	*“Well one of the challenges is the patronage, if it is high, they may not have time for all the patients, that is one. Then, there may be a mis-diagnosis, and that one if they mis-diagnose the patient, they will surely give wrong medication …“***P8/FGD/HCP** *“Access to equipment's, finance …“***P11/FGD/HCP**
	Funds for patients	*“Well, financial power of the patient, because for somebody who is just coming with probably, what can take basic … maybe just buy ordinary diclofenac, another person coming up, ready to take up other supportive drugs and all the rest”.***P2/FGD/CP**
	Health seeking behavior of patients.	*“… the attitude of our patient towards seeking appropriate care, and funny enough you see some of these patients, especially these ones that are not so cooperative, they get a prescription from somewhere instead of them to go back, may be complete the investigation, go for further review, they will carry the prescription from one pharmacy to the other …“***P2/FGD/HCP** *“… the level of cooperation that a patient who is actually involved wants to give, because in Africa here, we believe a lot of things could be the reason why a person is feeling a particular way, so when you are trying to give a suggestion or contribution based on your knowledge as a scientist, you might get some opposition. Culture also determines, culture determines is also a challenge, then sex, sex of the patient you are actually dealing with”.***P11/FGD/CP**
Recommendations for improving care.	Health education and promotion	*“So, you need to have a lot of health education and promotion for the patient, the care givers, families, …so, if a patient has low social support or if an elderly is living alone, you need to carry along the care givers of the elderly, so that as you are talking to the elderly patient, you carry along their care givers, the family, they know what to do, because if there are some adjustments they need to make”***P8/FGD/HCP** *“The other thing I think is important when it comes to pharmacist is that continuous medical education. I know they do a lot of courses here and there, they have a basic understanding from their curriculum in school, as we know everything revolves every time, new development, new modalities of treatment, new standard of operation, I believe in everybody in every discipline to improve herself.”***P2/FGD/HCP**
	Health system strengthening	*“If we have NHIS that will cover these, people might in one way seek professional care …”* *“… if referral is best for the patient, referral is a good thing to do, every patient will get that benefit of a referral system”***P2/FGD/HCP**
Guidelines for the management of osteoarthritis.	Availability and use of guidelines in healthcare facilities	*“There are guidelines. I will say more general guideline in assessment, more in the objective assessment, we look more specifically into … regarding guidelines, in physiotherapy we do conservative management right and the guideline is directed towards assessment and management in the care of patient with OA, so we usually use a bio-psychosocial approach, which I believe other participants will be able to go in-depth on”.***P2/FGD/HCP** *“we need the guidelines, stipulating the things they should watch out for, especially the level of pain, people should not be made to bear pain …” “Also, to advice patients to see a physiotherapists if we are lucky to convince them with a guideline … I will not say physiotherapist alone now, other specialty, including ehmnn., even if you have to see a GP first, general practitioner first”.***P5/FGD/HCP** *“so I want to belief that it is a lot that a lot has to be done to make sure everybody is on the same page what do I have to lose or gain rather, when I have to hold on to patient and be treating them on and on. When I know there are people that are better qualified to do some of those things, that is my submission”.***P6/FGD/HCP**

Participant (P).

Community Pharmacist (CP).

Healthcare Practitioner (HCP).

Focus Group Discussion (FDG).

Upon analysis of the FGDs, five main themes were identified: assessment and diagnosis of OA, care navigation and current management of OA, community pharmacies as the first point of call, barriers to management of OA in low resource settings, and guidelines for management of OA. Themes are described below, and additional data is presented in Table 2 (overview of themes with associated quotes).

### 3.1 Theme 1: assessment and diagnosis of OA

Rightly, history taking, physical examination, and referral to healthcare specialties were reported as key to diagnosing OA and providing appropriate treatment to patients. However, these were perceived as directly linked to proficiencies and skills of the health professional being consulted, as well as patient socioeconomic status (affordability of further tests and consultations). Investigative procedures, including radiography (X-rays, MRI) and blood tests, tend to drive the diagnosis and assessments, which vary and are often subject to affordability by patients. Investigations also provided assurance and confidence to health professionals to validate their diagnosis and assessment.

*“After taking a good history, you also examine the patient. The next line of action is now to do investigation, to confirm your suspicions. And in investigation, the most important is radiological investigation, starting with the plain x-ray. After that you can move to further investigation depending on your line of suspicion, whether you need to do a CT or an MRI. Other than the radiologically investigation, you do some blood work- “EMR value”, rheumatoid factor, full blood count, by the time you have done all these, you will be able streamline your diagnosis, typically to what the patient has”.***P4/FGD/HCP**

### 3.2 Theme 2: care navigation and current management of OA

#### 3.2.1 Guidelines for the management of OA

Participants allude to awareness/existence of guidelines for managing OA joint pain but knowledge of the evidence-base-recommendations within the guidelines could not be ascertained in the FGD. Some participants expressed concern that there is non-conformity with stipulated guidelines for the management of OA, although there is actually no clinical practice guideline locally adapted and agreed across multidisciplinary health professions for use in Nigeria or West Africa.

*We do diagnosis, so often we get referral from the medical consultant and the patient get referred to us. We do our own medical diagnosis. We follow clinical guidelines, specific guidelines for the management of different OA*. **P2/FDG/HCP**

*“Well, there is definitely a guideline that is adopted in Nigeria, but I will not say I know it step by step or everything that is entailed in the guideline …”***P10/FGD/CP**

*“Concerning the guidelines we follow, I can't say much about that, am sorry to say …”***P5/FGD/HCP**

#### 3.2.2 Theme 2.2: care pathway/navigation

**Fig. 1:**
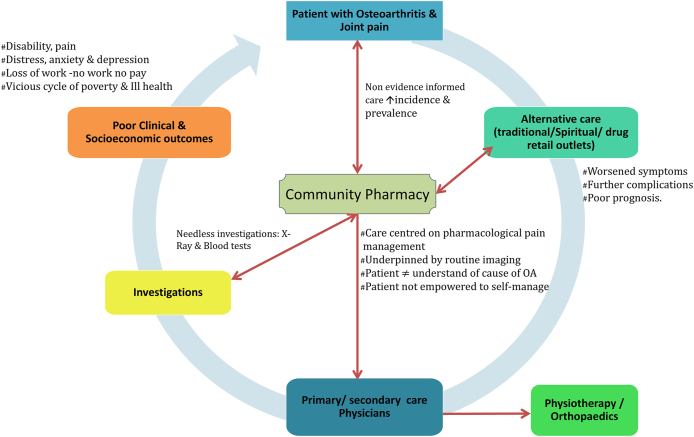
Current usual care pathway of care for people with OA in Nigeria.

Recounted initially from community pharmacists FGD, and later validated with the multidisciplinary health professionals FGD, Fig. 1 depicts the current usual care pathway of care for people with OA in Nigeria. For many of the health professionals who participated in the FGD, pharmacological approaches for pain relief tend to underpin management of OA. There was no formally defined, agreed local care pathway established for use in the Nigerian healthcare system. Large variations therefore existed in practice and use of pharmacological and non-pharmacological approaches to management. Treatment decisions were largely influenced by patient expectations/demands for medications to address pain symptoms and also available expertise at time of consultation (in case of community pharmacies where technicians often attend patients) and affordability of investigative procedures, medications, physical aids and supplements.

*Well, it depends on the level of the arthritis, in some cases, it’s just a physiotherapist that they need, in some cases, they need what’s it called, orthopedic doctor, so it depends on the level, maybe the reason why I talk like this is because one, most of the patients when they come, the first thing that brings them to you is pain, and they just want something to take care of the pain, and once you see that okay, with the level this one is going, it is not just about the pain. Sometimes I refer them to the lab myself, to do some test, so based on the outcome of the test, will help me to know where I am going to refer the patient to, if he’s just going to see an orthopedic or he’s going to see a physiotherapist.***P5/FDG/CP**

Health professionals believed socio-economic status has a role to play in prescribed management plan and patient's response to treatment. Therapeutic goal of relieving pain was mentioned among all participants as the key to the management of OA.

*“Now if this patient is a known patient, and I know is someone who can afford, because affordability is another thing we are looking at, so aside taking medications you want to follow up with the physiotherapist, one I have a contact with, I refer them to, and I am sure that they will also go to, because sometimes you will refer patients and the person will not go”*. **P10/FDG/CP**

Some health professionals are aware of but do not routinely recommend non-pharmacological approaches such as diet modification, exercise, social support due to patient beliefs or expectations for pain medications, and difficulty accessing specialist care.

*“yes, many times, and like you said, more women than men, emmm, generally, they come in complaining of pain, so their main aim when they come in is to get anything that will get away the pain, that is what most time they are concerned about. So, you want to ensure, anything you want to do to manage them has to first of all take care of the pain, anything you do that doesn’t take care of the pain, they are not interested”*. **P11/FDG/CP**

### 3.3 Theme 3: multidisciplinary care and community pharmacies as first point of call

Participants highlighted that community pharmacies and drug retail outlets are the popular first point of call by many patients seeking help and relief from aches and pain, including those with symptoms of OA. Participants agreed that the role of community pharmacists is vital and cannot be overlooked. Described as gatekeepers, participants shared perspectives on the support needs of community pharmacies involved in the care of people with OA. Training and upskilling on evidence-informed management of OA and joint pain were prioritised for community pharmacy teams as well as for other healthcare professionals to enhance multidisciplinary team working, effective referral pathway and to ultimately enhance patient care.

*“Community pharmacists, they are a part, a very functional part of the medical health care system, and we are embodied with all the things that has to do with drugs, that's why I said, before, at times before you even give out drugs, you ask some questions,..so that you don't aggravate other problems”.***P6/FGD/CP**

*“In Nigeria most patient see community pharmacist first before coming to the clinic and we cannot deny that. We need to work together with them. So, I think we need to empower community pharmacists because a patient goes to them first. The guidelines, the things they should watch out for, and do, especially the level of pain, people should not be made to bear pain …”***P5/FGD/HCP**

### 3.4 Theme 4: barriers to management of OA

Participants listed various challenges that hinder effective management of OA in Nigeria which they felt were common to those in similar low-resource settings across West Africa: OA in the presence of other co-morbid long-term conditions, patient cultural beliefs, poor health seeking behavior and attitude, pain threshold, multi-pharmacy visits, statutory referral powers and systems – professional jurisdictions, roadside patronage, patient self-medication and poly-pharmacy (including local herbs), non-adherence/noncompliance to prescribed medication, health systems structures and resources (including availability of equipment and drugs), lack of access to health education and promotion, out-of-pocket expenses, poor interdisciplinary and interpersonal collaboration/interaction, and poor practices.

*“Access to equipment's, finance, and the level of cooperation that a patient who is actually involved wants to give, because in Africa here, we believe a lot of things could be the reason why a person is feeling a particular way, so when you are trying to give a suggestion based on your knowledge as a scientist, you might get some opposition. Culture also determines, culture determines is also a challenge, then sex, sex of the patient you are actually dealing with”*. **P11/FGD/CP**

*“Well, financial power of the patient, because for somebody who is just coming with probably, what can take basic … maybe just buy ordinary diclofenac, another person coming up, ready to take up other supportive drugs and all the rest”.***P2/FGD/CP**

*“… the urban community pharmacists, they do not have much time, they are not easily accessible, some are busy in their office dispensing drugs etc., doing all of those things”.***P8/FGD/HCP**

*“The other aspect I want to mention is the aspect of making sure that we have a two way understanding of having collaborative care, such that when patients are referred, and they are also referred back to the pharmacists (with feedback and plan for monitoring) that will be an encouragement for them and for me …”***P6/FGD/HCP**

“*… if the pharmacist in their own wisdom now decide that Ha, Madam or Oga (***referring respectively to female/Male customer with OA***), that please oh, this pain you have been carrying it for so long you should go back to the hospital, they will go somewhere else, to the extent of buying by the road side what they called 'akapo' (***referring to “Akapo” - combination of many analgesics and other drugs sold together by patent medicine stores/chemists for pain and other illnesses***), this combination of analgesic that they take, so a lot need to be done when it comes to health education and promotion by the nurses then the social workers … the other problem we have in this environment is that they have to pay out of pocket “.*

*”… the patient psychic is important and then accessibility, like I mentioned there was a time I was posted to a xxx community, hardly will you see a specialist there, no physio, even the doctors … the system, it feels hopeless”***P2/FGD/HCP**

### 3.5 Theme 5: recommendations for improving OA care

Health professionals were keen to improve practice through provision of evidence-informed care but noted lack of contextual evidence and health systems infrastructure to inform optimised care programs (Box 2). As current provisions supports basic medical care packages, extending state health insurance package/schemes to support specialist care (including physiotherapy) for patients were suggested to address health seeking behavior and access to treatment. Continuous education for all stakeholders, i.e. patients, care givers and the healthcare professionals (including training specific to OA and musculoskeletal health) was highlighted to be very important. Also, it was felt that a well-structured referral system aided by an agreed local care pathway and treatment guidelines will support appropriate professional help as needed across multidisciplinary teams.

“… *Then, there should be room for interdisciplinary or interpersonal collaboration like we are having now … When will now come together as different discipline, different specialty and we have interaction we be able to rob mind, then will be able to learn from each other …“*

*We are doing a lot in healthcare, especially community pharmacists, this is the first of its kind that anybody is putting the spotlight on community pharmacists, we wish to have more of this collaborative effort, and actually, moresupport being given to the community pharmacists practice, so it becomes also as em, competitive as other aspect of the health care, yes thank you.***P8/FDG/CP**

Box 2 **Summary of recommendations**

**Figure:**
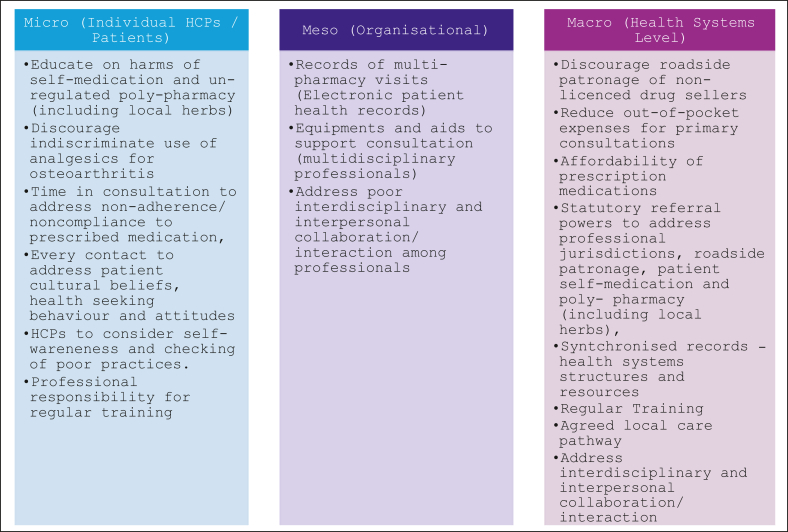

Alt-text: Box 2

## 4 Discussion

This study investigated community pharmacists' and other healthcare professionals' perspective on the resources and capability needed to manage OA in line with evidence-based recommendations, and to work together in caring for people with OA and joint pain. We find that Nigeria's osteoarthritis care pathway is fragmented, involves overuse of pharmacological approaches which are not in line with international guidelines/evidence-based recommendations. The FDG confirmed that in low-resource settings like Nigeria, community pharmacy teams being a usual first point of call, have a crucial role in evidence-informed care for people living with OA. People visit community pharmacies for medications whenever relief from pain and other symptoms are needed and when traditional self-care approaches have failed. However, there are no agreed/established care pathway for the management of OA by healthcare professionals in Nigeria. Hence, there is a wide variation in practice and for many teams, current practices in OA care are not in line with core guideline-recommended approaches. This is in line with previous reports that have indicated a poor consensus in the management of OA in Nigeria [24,25] though there is limited contextual research evidence on what works and how best in these settings. Specifically, pharmacological approaches to management of OA were dominant across professional practice though not in line with current evidence. A major finding was the need to develop a local care pathway for the management of OA, specific to African context based on current evidence. This view was also the position of Eyles et al. [26], who reported the complexity that may arise because of not having an approved indigenous guideline for managing OA.

Additional investigations (such as CT scan, rheumatoid factor, MRI, X-ray, and other blood tests) after taking patient history was central to assessment, diagnosis and management of OA in Nigeria. This is contrary to current evidence and international guideline recommendations [25]. Whereas, where healthcare professionals are well trained, and well-informed on OA assessment and diagnosis, further investigations including X-ray investigations which have resource constraints are not always needed and have no significant effect on evidence-based management of OA. This is particularly important in low-resource settings like Nigeria. Overuse of investigations increases out-of-pocket expenses for patients, leads to delayed treatment, detracts from core recommended approaches for management and can also take limited investigation resources away from those who need it most.

Our findings are in line with Cunningham et al. [27] who classified barriers to OA management as occurring at the micro level, involving the individuals (patients and HCP), meso level (organizational) and macro level, which involves the entire health system. At the micro-level, inability to get proper information from patients was a major barrier, as well as the inability of many patients (usually low-health literacy) to adhere to prescribed treatment or self-management plans. Furthermore, the socio-economic impact of out-of-pocket expenses affects access to, and affordability of appropriate treatments. At the meso-level, lack of up-to-date knowledge about OA among community pharmacists and other healthcare professionals was also highlighted. There is an acute skills gap in understanding of pain/other OA symptoms, assessment, diagnosis and core-recommended approaches to management, specifically for community pharmacists and health professionals in primary care or community settings who are usually the patient's first point of entry to the healthcare system. Given shortage of skilled HCPs in Nigeria, (e.g. patient/clinician ratio 0.047 physiotherapists per 1000 of the population) [28] partially due to brain drain – migration of skilled health workers to HICs, high population needs and consistently low health-care investments, training is needed for community pharmacists and rural health workers who could be more involved in the management of OA. This is in line with new propositions and research findings in the UK suggesting a model of care including community pharmacies as part of core multidisciplinary healthcare team involved in the management of OA and joint pain [14]. With adequate training and upskilling, the extended role of community pharmacies in OAmanagement could include: a subjective assessment, provision of information explaining the joint problem and its treatment, medication management and supported self-management [15].

Perception of community pharmacists as most accessible and popular first point of call by people seeking “non-urgent”/non-emergency care in Nigeria also resonated with our findings [29]. In Nigeria, community pharmacists serve as a crucial resource for primary healthcare and are a popular point of contact for health-related concerns [30]. Nigerian community pharmacists provide comprehensive health care services, including minor illness treatment, medication counseling, health education, point-of-care tests, immunization, and family planning. A lot of community pharmacist-led interventions have significantly improved disease prevention and drug-related care outcomes in primary health care settings [31]. However, the role of community pharmacists in adequately managing OA has not been well documented. Participants in our FGD believed most community pharmacists do make referrals (when necessary) to general physicians, rather than specialists specifically trained to manage patients with OA, such as physiotherapists, orthopedics or even dieticians (in case of need to control weights and proper diets). Moreover, fear of misdiagnosis and lack of commitment to OA management due to other pressing roles of the community pharmacists are some other factors that were mentioned as hindering the community pharmacists from actively participating in the management of OA. Lack of feedback mechanism was also mentioned as a factor that discourages community pharmacists from active participation in OA management. Generally poor collaboration among healthcare professionals was mentioned across all participants and in the two focus groups. This finding is similar to Weinberg et al. (2011) and Sullivan et al. (2019) [32,33] who found rigid hierarchy of healthcare occupations (common in Nigerian health system) is a major constraint to patient-centered care.

Participants felt that an optimised model of care needs to include social support (community-based) and health education for patients as part of routine care and sustainable self-management infrastructure. They posit it would be helpful if OA patients have a good understanding of their condition, so that they can self-manage but the time and resources to do this in busy practices was simply not feasible.

### 4.1 Future research focus on OA in West Africa and other LMICs

**Fig. 2:**
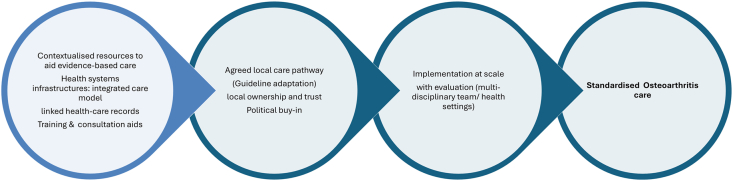
Strategies to address support needs of HCPs and improve care for OA in Nigeria.

In this study, perceived use of guidelines in the assessment, diagnosis and management of patients are subjective and does not follow core recommendations based on evidence and standard international practice. Our study findings indicated little health systems investment/evidence-based structure to facilitate quality musculoskeletal care. Briggs et al. also highlighted the need for global guidance to strengthen musculoskeletal health systems, emphasizing its often-overlooked priority despite its significant contribution to global disability [34]. Future implementation of evidence-based recommendations for OA needs to target standardization of care across multi-disciplinary professional teams. An important target to achieve this would be co-development and adoption of a local care pathway and clinical practice guideline sensitive to the socio-cultural context and the local health system (See Fig. 2 for suggested step-wise strategy). Multi-stakeholder collaboration is needed to drive investment into research and care of people with OA and musculoskeletal pain conditions as a matter of priority given its implication on the health of the workforce and socio-economic impact.

Though our study findings have implications for future research and practice across many similar settings in Africa and low-resource contexts, it is interpreted first within Nigerian context and methodological approach. Nigeria is a vast country with six geo-political zones, but our participants were recruited from South-West Nigeria. Homogeneity and heterogeneity among focus group participants is an issue for consideration, hence our first focus group was solely among community Pharmacists (varying socio-demographics including ethnicity) while the 2nd focus group among multi-disciplinary healthcare professionals with varying socio−demographic and ideological diversity among participants, enhanced multiple perspectives and reflections on different experiences. Within the FGDs, all participants had the opportunity to express themselves, contribute to discussions and asked each other questions thereby influence the collective outcomes. Participants in the 2nd FGD particularly praised the efforts in this project to bring multidisciplinary professionals together. For both FGDs, we took steps to achieve sufficient depth and breadth of an understanding of the experiences of healthcare professionals who consented to be part of our study. Sampling from other regions in Nigeria may have extended our understanding of concepts relating to evidence-based care of OA that were presented in this manuscript.

## 5 Conclusion

Our study highlights a significant gap in care and expresses needs for knowledge, and capacity building of health professionals in managing joint pain. Community pharmacies as a usual first point of call for patients present an opportunity for education and self-management advice, supporting patients with OA. However, future efforts to optimize services must be contextual and should consider the identified barriers and facilitators in this study. evidence-based research in efforts to align with global best practices for management of patients with OA.

## Author contributions

OB conceived and designed the study with input from the JIGSAW-A study team and patient representatives. OB, TO, OA, and IA were involved in data collection and first stage data analysis. OB and EA co-drafted the manuscript. All authors were involved in interpretation of findings, contributed to drafts, and approved final version of the manuscript.

## Ethics approval

Ethical approval has been granted by the University of Ibadan, Research Ethics Committee and Keele University FMHS Research Ethics committee (UI/SSHEC/2022/0023; KU0420).

## Funding

This study was supported by grants from The Global Awards for Advancing Chronic Pain Research (ADVANCE) Pfizer Incorporated, New York (Grant Award No: 70077279).

The funders had no role in the design and conduct of the study; collection, analysis, and interpretation of the data; preparation, review, or approval of the manuscript; or decision to submit the manuscript for publication.

## Declaration of competing interest

Authors do not have any conflict of interest to declare.

## References

[1] Bannuru R., Osani M., Vaysbrot E., Arden N., Bennell K., Bierma-Zeinstra S. (2019). OARSI guidelines for the non-surgical management of knee, hip and polyarticular osteoarthritis. Osteoarthritis cartilage journal.

[2] Tiffany Gill, Manasi Mittinty, March Lyn (2023). Global, regional, and national burden of other musculoskeletal disorders, 1990–2020, and projections to 2050: a systematic analysis of the Global Burden of Disease Study 2021. The Lancet Rheumatology.

[3] Woolf A.D. (2015). Global burden of Osteoarthritis and musculoskeletal diseases. BMC Muscoskelet. Disord..

[4] Yucesoy B., Charles L.E., Baker B., Burchfiel C.M. (2015 Jan 1). Occupational and genetic risk factors for Osteoarthritis: a review. Work.

[5] Bitton Ryan (2009). The economic burden of osteoarthritis. Am. J. Manag. Care.

[6] Mujtaba S.H., Gazerani P. (2024). Exploring the role of community pharmacists in pain management:EnablersandChallenges. Pharmacy.

[7] Moseng T., Vliet Vlieland T.P.M., Battista S., Beckwée D., Boyadzhieva V., Conaghan P.G., Costa D., Doherty M., Finney A.G. (2024). EULAR recommendations for the non-pharmacological core management of hip and knee osteoarthritis: 2023 update. Ann. Rheum. Dis..

[8] Andrew J., Peter R., David J., Tom A., Rodney S. (2022). Osteoarthritis management: does the pharmacists play a role in bridging the gap between what patients actually know and what they ought to know? Insight from a national online survey. International public participatory health care and health policy journal.

[9] Thapa P., Kc B., Gyawali S., Leong S.L., Mohamed Ibrahim M.I., Lee S.W.H. (2024). Effectiveness of community pharmacist-led interventions in Osteoarthritis pain management: a cluster-randomized trial. Res. Soc. Adm. Pharm..

[10] Owoyemi T., Alonge I., Adetunji O., Ogbu E., Ogunbanjo A., White S., Adebajo A., Mallen C., Babatunde O.O., Dziedzic K. (2024 Nov 29). Everyday living with osteoarthritis in the global South: a qualitative focus group inquiry in Nigeria. Osteoarthr Cartil Open.

[11] Babatunde OO, Adetunji O, Alonge I, Owoyemi T, Ayinmode E, Ogunbanjo A (2025). Process and feasibility of implementing guideline recommendations for the care of osteoarthritis in West Africa. BMJ Global Health.

[12] Allison T., Peter S., Jonathan C. (2007). Consolidated criteria for reporting qualitative research (COREQ): a 32-item checklist for interviews and focus groups. Int. J. Qual. Health Care.

[13] Krueger R.A., Casey M.A. (2000).

[14] Babatunde O.O., Cottrell E., White S. (2024). Co-development and testing of an extended community pharmacy model of service delivery for managing osteoarthritis: protocol for a sequential, multi-methods study (PharmOA). BMC Muscoskelet. Disord..

[15] Simkins J., Holden M.A., Babatunde O. (2024). Exploring the potential extended role of community pharmacy in the management of osteoarthritis: a multi-methods study with pharmacy staff and other healthcare professionals. Muscoskel. Care.

[16] Eyles J.P., Sharma S., Telles R.W., Namane M., Hunter D.J., Bowden J.L. (2022 Jan 24). Implementation of best-evidence osteoarthritis care: perspectives on challenges for, and opportunities from, low and middle-income countries. Frontiers in rehabilitation sciences.

[17] Hunter D.J., Lo G.H. (2008 Aug 1). The management of osteoarthritis: an overview and call to appropriate conservative treatment. Rheum. Dis. Clin. N. Am..

[18] Ayanniyi O., Egwu R.F., Adeniyi A.F. (2017 Jun 1). Physiotherapy management of knee osteoarthritis in Nigeria—a survey of self-reported treatment preferences. Hong Kong Physiother. J..

[19] Castleberry A., Nolen A. (2018). Thematic analysis of qualitative research data: is it as easy as it sounds?. Currents in pharmacy teaching and learning.

[20] Atkins L., Francis J., Islam R. (2017). A guide to using the Theoretical Domains Framework of behavior change to investigate implementation problems. Implement. Sci..

[21] Corbin Juliet, Strauss Anselm (2012).

[22] May C., Mair F., Finch T., MacFarlane A., Dowrick C., Treweek S. (2009). Development of a theory of implementation and integration: normalization process theory. Implementation Sci. journal.

[23] Braun V., Clarke V. (2006). Using thematic analysis in psychology. Qual. Res. Psychol..

[24] Long H., Liu Q., Yin H. (2022). Prevalence trends of site-specific osteoarthritis from 1990 to 2019: findings from the Global Burden of Disease study 2019. Arthritis Rheum..

[25] National Clinical Guideline Centre (2014).

[26] Eyles J.P., Bowden J.L., Redman S., Melo L., Dorio M., Hunter D.J. (2020). Barriers and enablers to the implementation of the Australian osteoarthritis chronic care program (OACCP). Osteoarthr. Cartil..

[27] Cunningham P.S., Ahern S.A., Smith L.C., da Silva Santos C.S., Wager T.T., Bechtold D.A. (2016). Targeting of the circadian clock via CK1δ/ε to improve glucose homeostasis in obesity. Sci. Rep..

[28] Odumodu I.J., Olufunlayo T.F., Ogunnowo B.E. (2020). Satisfaction with services among attendees of physiotherapy outpatient clinics in tertiary hospitals in Lagos State. J Patient Exp.

[29] Bamgboye A.O., Hassan I.A., Fatoye E.O., Ozuluoha C.C., Folami S.O., Uwizeyimana T. (2024 Feb 15). Enhancing care transition performance of community pharmacies in Nigeria. Health Sci. Rep..

[30] Ihekoronye M.R., Osemene K.P. (2022). Evaluation of the participation of community pharmacists in primary healthcare services in Nigeria: a mixed-method survey. Int. J. Health Pol. Manag..

[31] Jakeman B., Logothetis S.J., Roberts M.H. (2020). Addressing latent tuberculosis infection treatment through a collaborative care model with community pharmacies and a health department. Prev. Chronic Dis..

[32] Weinberg D.B., Cooney-Miner D., Perloff J.N., Babington L., Avgar A.C. (2011). Building collaborative capacity: promoting interdisciplinary teamwork in the absence of formal teams. Med. Care.

[33] Sullivan J.L., Weinburg D.B., Gidmark S., Engle R.L., Parker V.A., Tyler D.A. (2019). Collaborative capacity and patient-centered care in the veterans' health administration community living centers. Int J Care Coord.

[34] Briggs A.M., Jordan J.E., Kopansky-Giles D. (2021). The need for adaptable global guidance in health systems strengthening for musculoskeletal health: a qualitative study of international key informants. glob health res policy.

